# Health Literacy and Medication Management in Chronic Kidney Disease

**DOI:** 10.3928/24748307-20170607-01

**Published:** 2017-07-31

**Authors:** Genevra Galura, Amy Barton Pai

Chronic kidney disease (CKD) is the progressive decline of kidney function, which may eventually lead to kidney failure requiring dialysis or transplant for survival (Kidney disease improving global outcomes [KDIGO] 2012 clinical practice guideline for the evaluation and management of chronic kidney disease). Among patients older than age 65 years, CKD care accounts for 20% of all Medicare spending or $50.4 billion in 2013 (United States renal data system [USRDS] 2016 annual data report). Adjusted mortality rates are also higher for Medicare patients with CKD than those without CKD (USRDS, 2016). Over one-half of patients with CKD have significant comorbid conditions (USRDS, 2016). Awareness of CKD and its consequences is poor, even in patients with a diagnosis of CKD. Less than 10% of survey participants with stage 1–3 CKD between 2001 and 2012 were aware that they had the disease (USRDS, 2016). When compared to hypertension or diabetes, awareness of CKD is much lower. Awareness among patients with hypertension and diabetes has been reported to be greater than 90% (Tuot et al., 2016).

## Medication-Related Problems are Common and Costly in Patients with CKD

Almost one-quarter of patients in the general population are nonadherent to prescribed medications or do not take their medications as directed (DiMatteo, 2004). This incurs an estimated $105 billion of avoidable costs to the United States health care system each year, with 69% of costs due to hospitalizations (Aitken & Valkova, 2013). Comparatively, the most recent estimate of research and development costs for a new pharmaceutical medication is $2.870 million, including postapproval costs (DiMasi, Grabowski, & Hansen, 2016). Thus, the cost burden to the US health system due to nonadherence to medications is far greater than the development of new medications. Patients with CKD are at high risk for nonadherence and other medication-related problems (MRPs), including use of drugs without documented indication, improper drug selection or dosing, and inappropriate laboratory monitoring (Cardone, Bacchus, Assimon, Pai, & Manley, 2010). Risk factors for nonadherence and other MRPs include older age, at least three concurrent disease states, higher number of medications, greater frequency of dosing, and medications that require frequent monitoring (Bedell et al., 2000; Cardone et al., 2010; Osterberg & Blaschke, 2005). Patients with CKD typically have these risk factors. End-stage renal disease (ESRD) patients receiving dialysis, for example, are prescribed an average of 12 medications (Manley et al., 2004). Effective cost containment has been shown by detecting and addressing MRPs. In one study, it was estimated that the US health care system could save $4 for each $1 spent addressing MRPs (Manley & Carroll, 2002).

## Improving Health Literacy is Fundamental to Improving CKD Awareness and Medication Adherence

Health literacy is defined as “the degree to which individuals have the capacity to obtain, process, and understand basic health information and services needed to make appropriate health decisions” (Centers for Disease Control and Prevention, 2010). The 2003 National Assessment of Adult Literacy found that only 12% of adults have proficient health literacy, and many disparities exist for different demographics (Kutner, Greenberg, Jin, Paulsen, & White, 2006). Notably, Black, Hispanic, American Indian/Alaska Native, and multiracial adults, adults age 65 years and older, and adults living below the poverty level all have lower levels of health literacy (Kutner et al., 2006). These demographic groups are also commonly affected by CKD (USRDS). The pooled prevalence rate across 20 studies of all stages of CKD has been reported as 25% (Taylor et al., 2017). Addressing health literacy is critically important because lower literacy has been associated with poorer health outcomes, including increased use of emergency services, hospitalizations, and all-cause mortality (Berkman, Sheridan, Donahue, Halpern, & Crotty, 2011). In patients with ESRD, lower health literacy has been associated with both lower kidney disease knowledge and higher mortality. A prospective cohort study using the Rapid Estimate of Adult Literacy in Medicine (REALM) found that each 10-point decrease in health literacy score in dialysis patients was associated with a 16% increase in risk of death (Cavanaugh et al., 2010).

Conversely, in a meta-analysis of patients with various disease states, a positive association between health literacy and medication adherence has been observed (Zhang, Terry, & McHorney, 2014). There are currently no data in the CKD population evaluating the relationship of health literacy with adherence. In patient populations similar to CKD, such as patients with hypertension and diabetes, there is some debate over whether health literacy and adherence are strongly associated. For example, no consistent link was found in a systematic review of health literacy and adherence to diabetes or cardiovascular medications in older adults (Loke, Hinz, Wang, & Salter, 2012). In diabetes medication adherence, however, health literacy has been shown to help explain racial disparities (Osborn et al., 2011). Health literacy was found to negate the effect of race on low medication adherence for African Americans.

In patients with diabetes and cardiovascular disease, low adherence has been associated with both increased risk of adverse events and increased costs. The out-of-pocket costs of diabetes medications can be a barrier to adherence, particularly for uninsured patients. One study estimated that if uninsured patients on at least one oral diabetes medication were adherent, 30% of them would spend over 10% of their income on health care (Miller, Sarpong, & Hill, 2015). For antihypertensive medications, risk of cardiovascular events increases with decreasing levels of adherence (Yang et al., 2016). Lower adherence also resulted in greater costs for emergency department visits and hospitalizations. In 2011, Koçkaya & Wertheimer developed calculations to estimate risk and cost reductions for outcomes including kidney disease, stroke, heart attack, and heart disease with perfect adherence to antihypertensive medications. This analysis found that for 100% adherent patients, there is a 37% direct risk reduction and a $5.9 billion direct cost reduction for development of kidney disease, specifically. Although the initial cost of medications is higher for achieving adherence, the potential for reductions in total costs of care with increasing adherence should be considered.

## Continued Challenges in Improving Health Literacy Around Medications in CKD

There are continued challenges to improving medication literacy and adherence, including the many steps required to educate patients on using medications correctly. Six steps have been proposed: fill, understand, organize, take, monitor, and sustain (Bailey, Oramasionwu, & Wolf, 2013). Patients who appropriately self-manage their medications must successfully complete each of these steps. Within each step of medication self-management there are one or more barriers for some patients, and thus room for potential interventions at each step, as described in **Table 1**.

For the “understanding” step, a common required source of information provided to patients is the US Food and Drug Administration (FDA) medication guide. These guides generally do not meet the Universal Precautions for Health Literacy (U.S. Department of Health & Human Services, 2010). In an analysis of 227 guides, only one was found to be appropriate for patients based on reading level and inclusion of important elements such as conclusions, summaries, or framing context (M. Wolf et al., 2012). Medication guides were not found to be useful across patient literacy levels and those with limited literacy also had the lowest levels of comprehension. This points to the need for revised FDA medication guides that incorporate patient-centered strategies such as patient focus groups and attention to plain language guidelines. Multiple prototypes using patient-centered strategies improved comprehension in all patients, including reducing the gap between those with marginal and adequate literacy by one-half (M. Wolf et al., 2014). However, improving medication guides alone may be insufficient to improve adherence and outcomes, as none of the prototypes resulted in an average comprehension rate over 80%. Therefore, innovative and multi-modal education initiatives (e.g., video, apps) should be developed and evaluated for effectiveness and impact on adherence in CKD (Kleinman, Shah, Shah, Phatak, & Viswanathan, 2017; Yeung et al., 2017).

Intervening at the “organizing” and “taking” medications steps **(Table 1**) are other ways that adherence and outcomes could be improved. Changing prescription drug labeling has been identified as one way to do this. Evidence-based best practices include simplifying language, improving typography, and standardizing symbols, although barriers due to state pharmacy laws may exist in implementing these practices (Bailey et al., 2013). Use of patient-centered labels with explicit instructions increased adherence specifically among patients with lower literacy and complex regimens (M. S. Wolf et al., 2016). In the general patient population, however, patient-centered labels did not improve adherence. However, the benefit to specific at-risk populations, which may include a large proportion of patients with CKD, can inform a targeted approach to this intervention. In terms of other patient-education materials such as those available online or in print from nonprofit or government resources, these are generally written well above the average patient's literacy level (Morony, Flynn, McCaffery, Jansen, & Webster, 2015). This lack of quality, usable materials makes it difficult for clinicians to provide patients with CKD with information to support adequate self-management of their disease and medications. Patient knowledge and comprehension of CKD as a disease state remain a challenge and may impact medication adherence, especially to medications that are added on as CKD progresses. There are multiple barriers that patients may face in understanding a CKD diagnosis including lack of symptoms, delayed discussions with health care providers, and feelings that there are no interventions that can change the course of CKD (Wright Nunes, Roney, Kerr, Ojo, & Fagerlin, 2016). Approaches to improve medication adherence should be integrated with early disease state education.

## The Role of the Pharmacist in Improving Literacy and Adherence

The provision of medication management services by pharmacists has been shown to identify and resolve MRPs and reduce medication costs and acute health care utilization (Cardone et al., 2010; Pai et al., 2009). In a 2-year randomized, controlled study in ambulatory hemodialysis patients, provision of medication management by a pharmacist identified over 500 MRPs (Pai et al., 2009). The most common problem was related to renal bone disease management with phosphate binders. More than one-half of the recommendations resulted in improvement of the inciting variable (e.g., lower serum phosphorus). Further development of systematic medication management services holds great potential to improve medication literacy, adherence, and outcomes in CKD (Pai et al., 2013). Systematic medication management services use standardized approaches such as care maps and algorithms. This ensures consistency and allows for the model to be reproduced in other sites. Phosphate binders are a class of medications with notably poor adherence rates. In hemodialysis patients, less than one-quarter remained totally adherent to phosphate binders over an 8-week study period (Van Camp, Vrijens, Abraham, Van Rompaey, & Elseviers, 2014). A Medicare cost-offset model showed that increased phosphate binder medication possession ratios and percent time in range for serum phosphorus reduced overall Medicare costs, most prominently for inpatient services (Ramakrishnan et al., 2014). A separate study found a novel approach to improving phosphate binder adherence in ESRD: self-affirmation (Ezeanolue et al., 2016). Before presenting “threatening” health information about phosphate control, patients were given either self-affirming or matched control questions. Although the evidence supporting self-affirmation was not overwhelmingly strong, this is a low-cost and high-reach intervention to improve serum phosphate control, and a way for pharmacists to improve adherence and decrease costs.

## Summary and Future Directions

CKD is a major health epidemic in the US. High costs for CKD patients result, in part, from patient's low health literacy and poor medication adherence. There are many missed opportunities for health care providers to improve patient education. These include creating more user-friendly medication information materials and encouraging use of existing, underutilized apps to support understanding and adherence to medications. Pharmacists, in particular, are well-positioned to intervene to improve adherence and outcomes for patients.

## Figures and Tables

**Table 1:** Steps to Improve Medication Adherence in Chronic Kidney Disease

**Steps**	**Challenges**	**Examples**	**Opportunities**
Fill prescription	Frequent dose adjustments of existing prescriptions cause patients to use wrong dosages. Prescription refills may not be synchronized. Gaps in time exist between prescribing and filling prescriptions. These gaps may be due to the use of multiple pharmacies, barriers in transportation, delays in mail orders of prescriptions, and insufficient pharmacy stock of infrequently used medications.	Most quantities of prescriptions for phosphate binders range from 180–480 tablets. Due to cost, pharmacies may not stock the entire quantity, leading to partial fills. Patients may not return to pick up remainder of fill.	Use reminder techniques such as putting prescription on refrigerator and optimizing use of patient portals or other electronic health records. Clinic to call the pharmacy 2–3 days after writing the prescription to close the loop.
Understand	In clinic Very little information is provided to patients during the visit, and this information typically is delivered only verbally. In pharmacy Information provided to patients about medications is not patient-centered or literacy appropriate. Counseling is required to be offered in most states, but it is often declined by patients or insufficient (Kimberlin, Jamison, Linden, & Winterstein, 2011). At home Many patients go to the Internet to find information but may not possess the skills to sift through what is valuable information.	A CKD stage 4 patient went to the emergency department for back pain and was prescribed 800 mg of ibuprofen every 6 hours. The patient had been counseled about kidney risks due to use of nonsteroidal anti-inflammatory drugs previously but did not feel empowered and deferred to the judgment of the emergency department physician. The patient experienced acute kidney injury from the ibuprofen. Phosphate binders work best when taken with the first bite of meals. However, patients often take them incorrectly after completing meals.	In clinic Provide good Internet and written resources. In pharmacy Develop and utilize materials that are literacy appropriate, in plain language that follows the AHRQ Universal Precautions for Literacy (http://www.ahrq.gov/professionals/quality-patient-safety/quality-resources/tools/literacy-toolkit/index.html). Improve prompts for counseling by using direct and open-ended questions. At home Provide good Internet resources in clinic that the patient can use to educate themselves at home.
Organize	CKD/ESRD patients are on many (average 12) medications and have a high medication regimen complexity index. Many patients have complex and frequent dosing regimens, so they must have medications in multiple locations (i.e., at home, in the car, at work). Administration directions vary by day for dialysis patients.	Patients using phosphate binders must carry a supply with them for whenever they eat out or have a snack.	Consolidate dosing regimens. Use apps such as MediSafe that can provide feedback to patients about their medication adherence (https://medisafe.com/). Use unit dose packaging that provides a single bag of medications to be taken at each administration time (e.g., PillPack, https://www.pillpack.com/).
Takes	Patients must remember to take the many doses of their medications. CKD patients have multiple medical encounters, which can hinder development of a routine. Many CKD patients have chronic gastrointestinal issues; when they don't feel well, they often don't take medications. Some patients have limited dexterity resulting in medication problems such as trouble drawing up doses of insulin.	When patients go out to eat, they may not remember to bring their phosphate binders with them.	Use apps and gamification to motivate or incentivize patients to take medications. Teach patients to plan their administration routines. Provide patients with the most common medications causing gastrointestinal side effects so that they can better guess and check what is making them feel unwell. Adjust regimens of challenging medications to the least common frequency and easiest administration routes.
Monitor	Many medications can result in many side effects. Patients with CKD may attribute adverse drug reactions to their disease state rather than medications.	ACE inhibitors commonly cause cough, but patients with CKD tend to be volume overloaded resulting in cough. Patients with CKD also tend to have many skin issues so they may not notice dermatologic side effects of medications.	Provide top three side effects or important monitoring parameters for each medication in easy, patient-centered formats. [See (Antonelli, 2016)]. Keep a log of potential side effects when starting a new medication, which could be done through an app or be an expanded role for pharmacists and pharmacies.
Sustain	Patients with CKD are on multiple medications. There is a high incidence of depression and nonadherence with progression of CKD.	With disease progression, medications are often added but rarely taken away. Deprescribing protocols should be initiated.	Streamlining, deprescribing, and consolidating medications can make complicated regimens easier to manage (Wright, Lovering, & Battistella, 2015). Provide better feedback about disease-state management and use incentives such as improved adherence will result in a delayed start of dialysis or an increased likelihood of a kidney transplant.

Note. ACE = angiotensin-converting enzyme; AHRQ = Agency for Healthcare Research and Quality; CKD = chronic kidney disease; ESRD = end-stage renal disease. Adapted from Bailey et al., 2013).

## References

[x24748307-20170607-01-bibr1] Aitken M. Valkova S. (2013). Avoidable costs in U.S. healthcare: The $200 billion opportunity from using medicines more responsibly. IMS Institute for Healthcare Informatics. Retrieved from http://www.imshealth.com/files/web/IMSH%20Institute/Reports/Avoidable_Costs_in%20_US_Healthcare/IHII_AvoidableCosts_2013.pdf

[x24748307-20170607-01-bibr2] Antonelli P. (2016, 11). Look again: Six designers take on some of the world's toughest redesign challenges. The New York Times. Retrieved from https://www.nytimes.com/interactive/2016/11/13/magazine/design-issue-redesign-challenge.html?_r=1

[x24748307-20170607-01-bibr3] Bailey S. C. Oramasionwu C. U. Wolf M. S. (2013). Rethinking adherence: A health literacy-informed model of medication self-management. Journal of Health Communication, 18(1), 20–30. 10.1080/10810730.2013.825672 PMC3814610 24093342

[x24748307-20170607-01-bibr4] Bedell S. E. Jabbour S. Goldberg R. Glaser H. Gobble S. Young-Xu Y. Ravid S. (2000). Discrepancies in the use of medications: Their extent and predictors in an outpatient practice. Archives of Internal Medicine, 160(14), 2129–2134. 10.1001/archinte.160.14.2129 10904455

[x24748307-20170607-01-bibr5] Berkman N. D. Sheridan S. L. Donahue K. E. Halpern D. J. Crotty K. (2011). Low health literacy and health outcomes: An updated systematic review. Annals of Internal Medicine, 155(2), 97–107. 10.7326/0003-4819-155-2-201107190-00005 21768583

[x24748307-20170607-01-bibr6] Cardone K. E. Bacchus S. Assimon M. M. Pai A. B. Manley H. J. (2010). Medication-related problems in CKD. Advances in Chronic Kidney Disease, 17(5), 404–412. 10.1053/j.ackd.2010.06.004 20727510

[x24748307-20170607-01-bibr7] Cavanaugh K. L. Wingard R. L. Hakim R. M. Eden S. Shintani A. Wallston K. A. Ikizler T. A. (2010). Low health literacy associates with increased mortality in ESRD. Journal of the American Society of Nephrology, 21(11), 1979–1985. 10.1681/ASN.2009111163 20671215 PMC3014012

[x24748307-20170607-01-bibr8] Centers for Disease Control and Prevention. (2010). What is health literacy. Retrieved from https://www.cdc.gov/healthliteracy/learn/index.html

[x24748307-20170607-01-bibr9] DiMasi J. A. Grabowski H. G. Hansen R. W. (2016). Innovation in the pharmaceutical industry: New estimates of R&D costs. Journal of Health Economics, 47, 20–33. 10.1016/j.jhealeco.2016.01.012 26928437

[x24748307-20170607-01-bibr10] DiMatteo M. R. (2004). Variations in patients' adherence to medical recommendations: A quantitative review of 50 years of research. Medical Care, 42(3), 200–209. 10.1097/01.mlr.0000114908.90348.f9 15076819

[x24748307-20170607-01-bibr11] Ezeanolue E. E. Nwizu C. Greene G. S. Amusu O. Chukwuka C. Ndembi N. Kozel T. R. (2016). Evidence that self-affirmation improves phosphate control in hemodialysis patients: A pilot cluster randomized controlled trial. Journal of Acquired Immune Deficiency Syndromes, 73(1), 117–121. 10.1097/QAI.0000000000001048 27144527 PMC4981538

[x24748307-20170607-01-bibr12] Kidney disease improving global outcomes (KDIGO). (2013). KDIGO 2012 clinical practice guideline for the evaluation and management of chronic kidney disease. Kidney International Supplements, 3(1), 1–150. Retrieved from http://kdigo.org/wp-content/uploads/2017/02/KDIGO_2012_CKD_GL.pdf

[x24748307-20170607-01-bibr13] Kimberlin C. L. Jamison A. N. Linden S. Winterstein A. G. (2011). Patient counseling practices in U.S. pharmacies: Effects of having pharmacists hand the medication to the patient and state regulations on pharmacist counseling. Journal of the American Pharmacists Association, 51(4), 527–534. 10.1331/JAPhA.2011.10012 21752776

[x24748307-20170607-01-bibr14] Kleinman N. J. Shah A. Shah S. Phatak S. Viswanathan V. (2017). Improved medication adherence and frequency of blood glucose self-testing using an m-health platform versus usual care in a multisite randomized clinical trial among people with type 2 diabetes in India. Telemedicine and E-Health. Advance online publication. . 10.1089/tmj.2016.0265 28328396

[x24748307-20170607-01-bibr15] Koçkaya G. Wertheimer A. (2011). Can we reduce the cost of illness with more compliant patients? An estimation of the effect of 100% compliance with hypertension treatment. Journal of Pharmacy Practice, 24(3), 345–350. 10.1177/0897190010389336 21676859

[x24748307-20170607-01-bibr16] Kutner M. Greenberg E. Jin Y. Paulsen C. White S. (2006). The health literacy of America's adults: Results from the 2003 National Assessment of Adult Literacy. U.S. Department of Education; National Center for Education Statistics; Institute of Education Sciences Retrieved from https://nces.ed.gov/pubs2006/2006483.pdf

[x24748307-20170607-01-bibr17] Loke Y. K. Hinz I. Wang X. Salter C. (2012). Systematic review of consistency between adherence to cardiovascular or diabetes medication and health literacy in older adults. Annals of Pharmacotherapy, 46(6), 863–872. 10.1345/aph.1Q718 22669802

[x24748307-20170607-01-bibr18] Manley H. J. Carroll C. A. (2002). The clinical and economic impact of pharmaceutical care in end-stage renal disease patients. Seminars in Dialysis, 15(1), 45–49. 10.1046/j.1525-139x.2002.00014.x 11874593

[x24748307-20170607-01-bibr19] Manley H. J. Garvin C. G. Drayer D. K. Reid G. M. Bender W. L. Neufeld T. K. Muther R. S. (2004). Medication prescribing patterns in ambulatory haemodialysis patients: Comparisons of USRDS to a large not-for-profit dialysis provider. Nephrology Dialysis Transplantation, 19(7), 1842–1848. 10.1093/ndt/gfh280 15128886

[x24748307-20170607-01-bibr20] Miller G. E. Sarpong E. M. Hill S. C. (2015). Does increased adherence to medications change health care financial burdens for adults with diabetes? Journal of Diabetes, 7(6), 872–880. 10.1111/1753-0407.12292 25817601

[x24748307-20170607-01-bibr21] Morony S. Flynn M. McCaffery K. J. Jansen J. Webster A. C. (2015). Readability of written materials for CKD patients: A systematic review. American Journal of Kidney Diseases, 65(6), 842–850. 10.1053/j.ajkd.2014.11.025 25661679

[x24748307-20170607-01-bibr22] Osborn C. Y. Cavanaugh K. Wallston K. A. Kripalani S. Elasy T. A. Rothman R. L. White R. O. (2011). Health literacy explains racial disparities in diabetes medication adherence. Journal of Health Communication, 16(3), 268–278. 10.1080/10810730.2011.604388 21951257 PMC3561717

[x24748307-20170607-01-bibr23] Osterberg L. Blaschke T. (2005). Adherence to medication. New England Journal of Medicine, 353(5), 487–497. 10.1056/NEJMra050100 16079372

[x24748307-20170607-01-bibr24] Pai A. B. Boyd A. Depczynski J. Chavez I. M. Khan N. Manley H. (2009). Reduced drug use and hospitalization rates in patients undergoing hemodialysis who received pharmaceutical care: A 2-year, randomized, controlled study. Pharmacotherapy, 29(12), 1433–1440. 10.1592/phco.29.12.1433 19947803

[x24748307-20170607-01-bibr25] Pai A. B. Cardone K. E. Manley H. J. St. Peter W. L. Shaffer R. Somers M. Mehrotra R. (2013). Medication reconciliation and therapy management in dialysis-dependent patients: Need for a systematic approach. Clinical Journal of the American Society of Nephrology, 8(11), 1988–1999. 10.2215/CJN.01420213 23990162 PMC3817900

[x24748307-20170607-01-bibr26] Ramakrishnan K. Braunhofer P. Newsome B. Lubeck D. Wang S. Deuson J. Claxton A. J. (2014). The economic impact of improving phosphate binder therapy adherence and attainment of guideline phosphorus goals in hemodialysis patients: A Medicare cost-offset model. Advances in Therapy, 31, 1272–1286. 10.1007/s12325-014-0170-4 25479935

[x24748307-20170607-01-bibr27] Taylor D. M. Fraser S. D. S. Bradley J. A. Bradley C. Draper H. Metcalfe W. Roderick P. J. (2017). A systematic review of the prevalence and associations of limited health literacy in CKD. Clinical Journal of the American Society of Nephrology. Advance online publication. . 10.2215/CJN.12921216 PMC5498363 28487346

[x24748307-20170607-01-bibr28] Tuot D. S. Zhu Y. Velasquez A. Espinoza J. Mendez C. D. Banerjee T. Powe N. R. (2016). Variation in patients' awareness of CKD according to how they are asked. Clinical Journal of the American Society of Nephrology, 11(9), 1566–1573. 10.2215/CJN.00490116 27340288 PMC5012470

[x24748307-20170607-01-bibr29] United States Renal Data System (USRDS). (2016). Annual data report 2016: Epidemiology of kidney disease in the United States. Bethesda, MD: National Institutes of Health, National Institute of Diabetes and Digestive and Kidney Diseases Retrieved from https://www.usrds.org/adr.aspx

[x24748307-20170607-01-bibr30] U.S. Department of Health & Human Services. Agency for Healthcare Research and Quality. (2010). AHRQ health literacy universal precautions toolkit. Retrieved from https://www.ahrq.gov/professionals/quality-patient-safety/quality-resources/tools/literacy-toolkit/index.html

[x24748307-20170607-01-bibr31] Van Camp Y. Vrijens B. Abraham I. Van Rompaey B. Elseviers M. (2014). Adherence to phosphate binders in hemodialysis patients: Prevalence and determinants. Journal of Nephrology, 27(6), 673–679. 10.1007/s40620-014-0062-3 24563270

[x24748307-20170607-01-bibr32] Wolf M. Bailey S. Serper M. Smith M. Davis T. Russell A. Lambert B. (2014). Comparative effectiveness of patient-centered strategies to improve FDA medication guides. Medical Care, 52(9), 781–789. 10.1097/MLR.0000000000000182 25119953

[x24748307-20170607-01-bibr33] Wolf M. King J. Wilson E. Curtis L. Bailey S. Duhig J. Lambert B. (2012). Usability of FDA-approved medication guides. Journal of General Internal Medicine, 27(12), 1714–1720. 10.1007/s11606-012-2068-7 22566170 PMC3509312

[x24748307-20170607-01-bibr34] Wolf M. S. Davis T. C. Curtis L. M. Bailey S. C. Knox J. P. Bergeron A. Wood A. J. J. (2016). A patient-centered prescription drug label to promote appropriate medication use and adherence. Journal of General Internal Medicine, 31(12), 1482–1489. 10.1007/s11606-016-3816-x 27542666 PMC5130952

[x24748307-20170607-01-bibr35] Wright A. Lovering S. Battistella M. (2015). Deprescribing: Is there a role in hemodialysis? Canadian Association of Nephrology Nurses and Technologies, 25(1), 21–23. 26882638

[x24748307-20170607-01-bibr36] Wright Nunes J. Roney M. Kerr E. Ojo A. Fagerlin A. (2016). A diagnosis of chronic kidney disease: Despite fears patients want to know early. Clinical Nephrology, 86(2), 78–86. 10.5414/CN108831 PMC5012189 27345185

[x24748307-20170607-01-bibr37] Yang Z. Howard D. Will J. Loustalot F. Ritchey M. Roy K. (2016). Association of antihypertensive medication adherence with healthcare use and Medicaid expenditures for acute cardiovascular events. Medical Care, 54(5), 504–511. 10.1097/MLR.0000000000000515 27078823

[x24748307-20170607-01-bibr38] Yeung D. L. Alvarez K. S. Quinones M. E. Clark C. A. Oliver G. H. Alvarez C. A. Jaiyeola A. O. (2017). Low-health literacy flashcards and mobile video reinforcement to improve medication adherence in patients on oral diabetes, heart failure, and hypertension medications. Journal of the American Pharmacists Association, 57, 30–37. 10.1016/j.japh.2016.08.012 27816544 PMC5507206

[x24748307-20170607-01-bibr39] Zhang N. J. Terry A. McHorney C. A. (2014). Impact of health literacy on medication adherence: A systematic review and meta-analysis. Annals of Pharmacotherapy, 48(6), 741–751. 10.1177/1060028014526562 24619949

